# A Practical Guide to Adjust Micronutrient Biomarkers for Inflammation Using the BRINDA Method

**DOI:** 10.1016/j.tjnut.2023.02.016

**Published:** 2023-02-13

**Authors:** Hanqi Luo, Jiaxi Geng, Madeleine Zeiler, Emily Nieckula, Fanny Sandalinas, Anne Williams, Melissa F. Young, Parminder S. Suchdev

**Affiliations:** 1 Hubert Department of Global Health, Rollins School of Public Health, Emory University, Atlanta, GA, United States;; 2 London School of Hygiene and Tropical Medicine, London, United Kingdom;; 3 University of Otago, Dunedin, New Zealand;; 4 Centers for Disease Control and Prevention, Atlanta, GA, United States

**Keywords:** micronutrients, inflammation adjustment, software, BRINDA

## Abstract

The Biomarkers Reflecting Inflammation and Nutritional Determinants of Anemia (BRINDA) research group was formed over a decade ago to improve the interpretation of micronutrient biomarkers in settings with inflammation. The BRINDA inflammation adjustment method uses regression correction to adjust for the confounding effects of inflammation on select micronutrient biomarkers and has provided important insights to micronutrient research, policy, and programming. However, users may face challenges when applying the BRINDA inflammation adjustment methods to their own data due to varying guidance on the adjustment approach for different biomarkers and the need to develop statistical programming to conduct these analyses. This may result in lost opportunities to have results of micronutrient data readily available during critical decision-making periods. Our research objectives are to *1*) provide an all-in-one summary of the BRINDA method in adjusting multiple micronutrient biomarkers for inflammation, *2*) evaluate whether malaria as a binary variable should be included in the BRINDA inflammation adjustment method, and *3*) present standardized and user-friendly BRINDA adjustment R package and SAS macro. This paper serves as a practical guidebook for the BRINDA inflammation adjustment approach and aids users to use the BRINDA R package and SAS to streamline their analyses.

## Background

Micronutrient deficiencies, also known as hidden hunger, have been said to affect 1 in 2 children and 2 in 3 women worldwide [1]. Micronutrient deficiencies contribute to poor growth, intellectual impairment, and increased risk of morbidity and mortality [2]. Having timely, valid data on micronutrient biomarkers is essential to justify, plan, and implement successful nutrition intervention programs [3]. One of the biggest challenges in interpreting micronutrient deficiencies is that inflammation can affect many micronutrient biomarkers and can therefore lead to incorrect diagnosis of individuals and to over- or underestimation of the prevalence of micronutrient deficiency in a population [4]. To improve the interpretation of micro-nutrient biomarkers in areas with high inflammation and to generate context-specific estimates of risk factors for anemia [4], a multiagency research group, the Biomarkers Reflecting Inflammation and Nutritional Determinants of Anemia (BRINDA) project, was formed in 2012 by CDC, Eunice Kennedy Shriver National Institute of Child Health and Human Development, and Global Alliance for Improved Nutrition, with the support from the Bill and Melinda Gates Foundation and under the secretariat of Emory University [5].

BRINDA’s primary goal is to improve global micronutrient assessment. Reliable data are essential for both policy making and practice to ensure that programs are appropriately designed and that nutritionally deficient populations are appropriately targeted. To date, BRINDA has compiled a global micronutrient database with 30 datasets on preschool-age children (PSC) (n = 20,000) from 26 countries and 24 datasets on nonpregnant women of reproductive age (WRA) (n = 56,000) from 23 countries. Using its rich data source, BRINDA published a series of papers to provide methods to adjust for inflammation on key micronutrient biomarkers, such as biomarkers for vitamin A, iron, folate, vitamin B-12, zinc, and vitamin D [4,6–13]. BRINDA’s effort to systematically address the role of inflammation in assessing micronutrient status has been recognized by the international community and is included in WHO guidelines [14] and CDC’s Micronutrient Manual and Toolkit [15].

The BRINDA inflammation adjustment method was developed to serve a wide range of users from the global nutrition community, including policy analysts who aid national nutrition strategy planning, researchers who study micronutrients, and field practitioners in charge of monitoring and evaluating micronutrient interventions. However, practically applying the BRINDA inflammation adjustment method to data can be complex. There is varying guidance on the adjustment approaches for different biomarkers. In addition, for the same iron and vitamin A bio-markers, according to the BRINDA’s initial publication [7], malaria status was added as a covariate and was also included in the WHO ferritin guideline [14]; however, later BRINDA papers on adjusting the role of inflammation on vitamin A, iron, and zinc [8, 9,11] did not recommend adding malaria as a binary covariate. Furthermore, users are required to develop statistical programming to conduct these analyses and must consider the time and technical skills in statistics and programming required to do so. Therefore, the time spent learning the BRINDA inflammation adjustment method and compiling correct programming codes may result in lost opportunities to have timely micronutrient data results during critical decision-making periods.

The need for global efforts to standardize guidance for micronutrient analysis and interpretation has been repeatedly emphasized by recent nutrition initiatives [3,16,17]. The objectives of this paper are to *1*) provide an all-in-one summary of the BRINDA inflammation adjustment method, *2*) evaluate whether malaria as a binary variable should be included in the BRINDA inflammation adjustment method, and *3*) present user-friendly, standardized BRINDA inflammation adjustment R package and SAS macro. It is important to mention that both the BRINDA inflammation adjustment method and this paper focus on WRA and PSC. However, the general principle of the BRINDA method can apply to other population groups.

## BRINDA Inflammation Adjustment Method

In the past few years, BRINDA has published a series of papers providing guidance on how to adjust micronutrient biomarkers, namely retinol binding protein (RBP) [8,9], serum retinol [8,9], serum ferritin [8], soluble transferrin receptor (sTfR) [10], serum zinc [11], serum and red blood cell (RBC) folate [12], serum B-12 [12], and vitamin D [13], using inflammation markers α-1-acid glycoprotein (AGP) and/or C-Reactive Protein (CRP) [6]. In this section, we will first present whether these micronutrient biomarkers should be adjusted for inflammation, and if so, by which inflammation markers (both AGP and CRP, only AGP, or only CRP). Second, we will summarize the procedure for applying the BRINDA inflammation adjustment method. Third, we will discuss whether including malaria as a binary variable in the BRINDA inflammation adjustment method is necessary. It has been widely recognized that serum and plasma values for micronutrient biomarkers such as RBP, retinol, ferritin, sTfR, zinc, folate, and B-12 have approximately the same values. Hereafter, we will use serum micronutrient biomarkers to refer to both plasma and serum micronutrient biomarkers.

## Which Micronutrient Biomarkers Should Be Adjusted for Inflammation?

**FIGURE 1.**
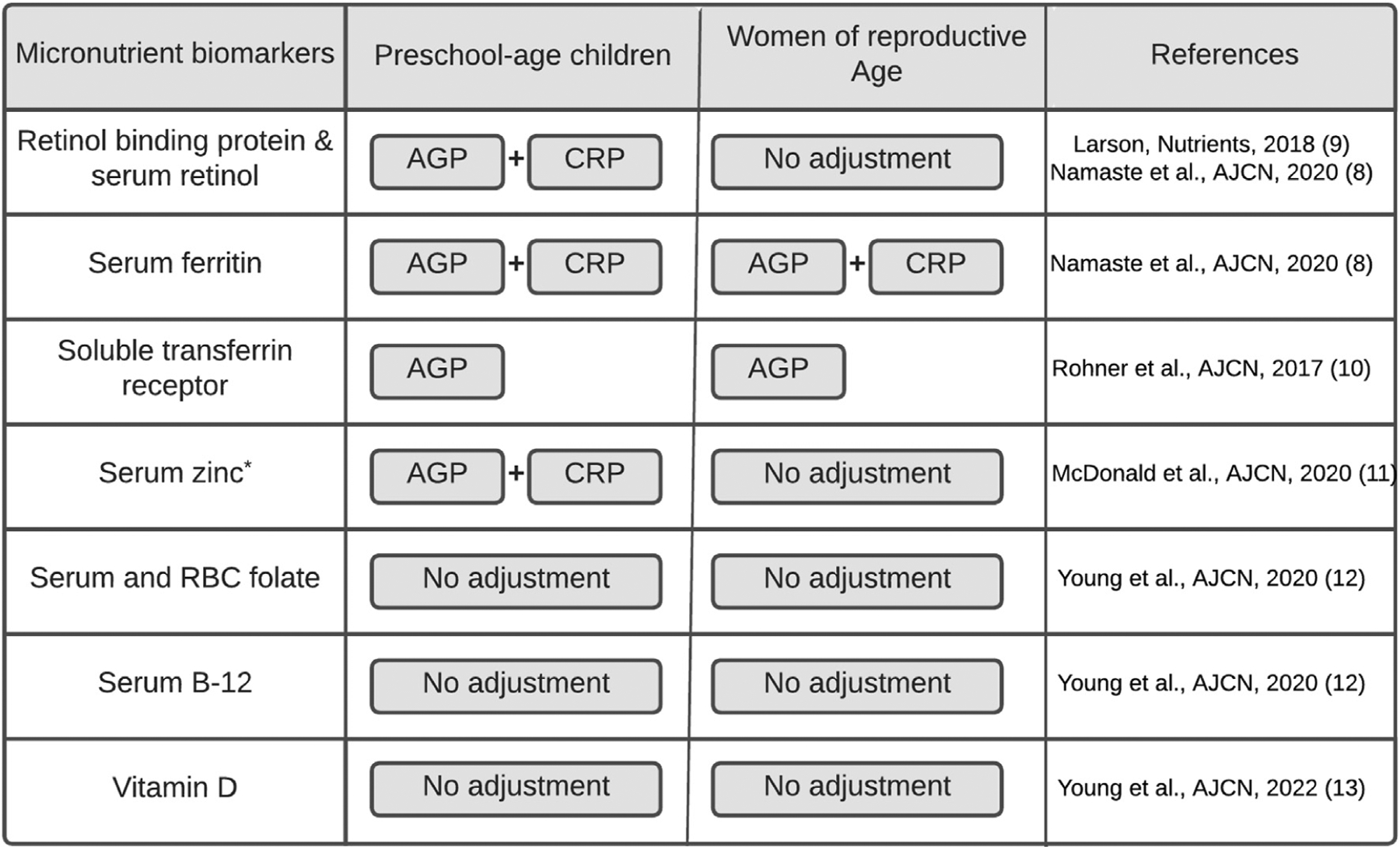
Inflammation markers used to adjust micronutrient biomarkers among preschool-age children and women of reproductive age based on the latest publications. If only one inflammation biomarker is available, for micronutrient biomarkers that need to be adjusted by both AGP and CRP, it is still recommended to use that inflammation marker to adjust for inflammation. AGP, α-1-acid glycoprotein. *Serum zinc needs to be adjusted by both AGP and CRP only if there is a negative (*r* < −0.1) and marginally significant (*P* < 0.1) Spearman correlation between serum zinc and either AGP or CRP in preschool-age children.

**FIGURE 2.**
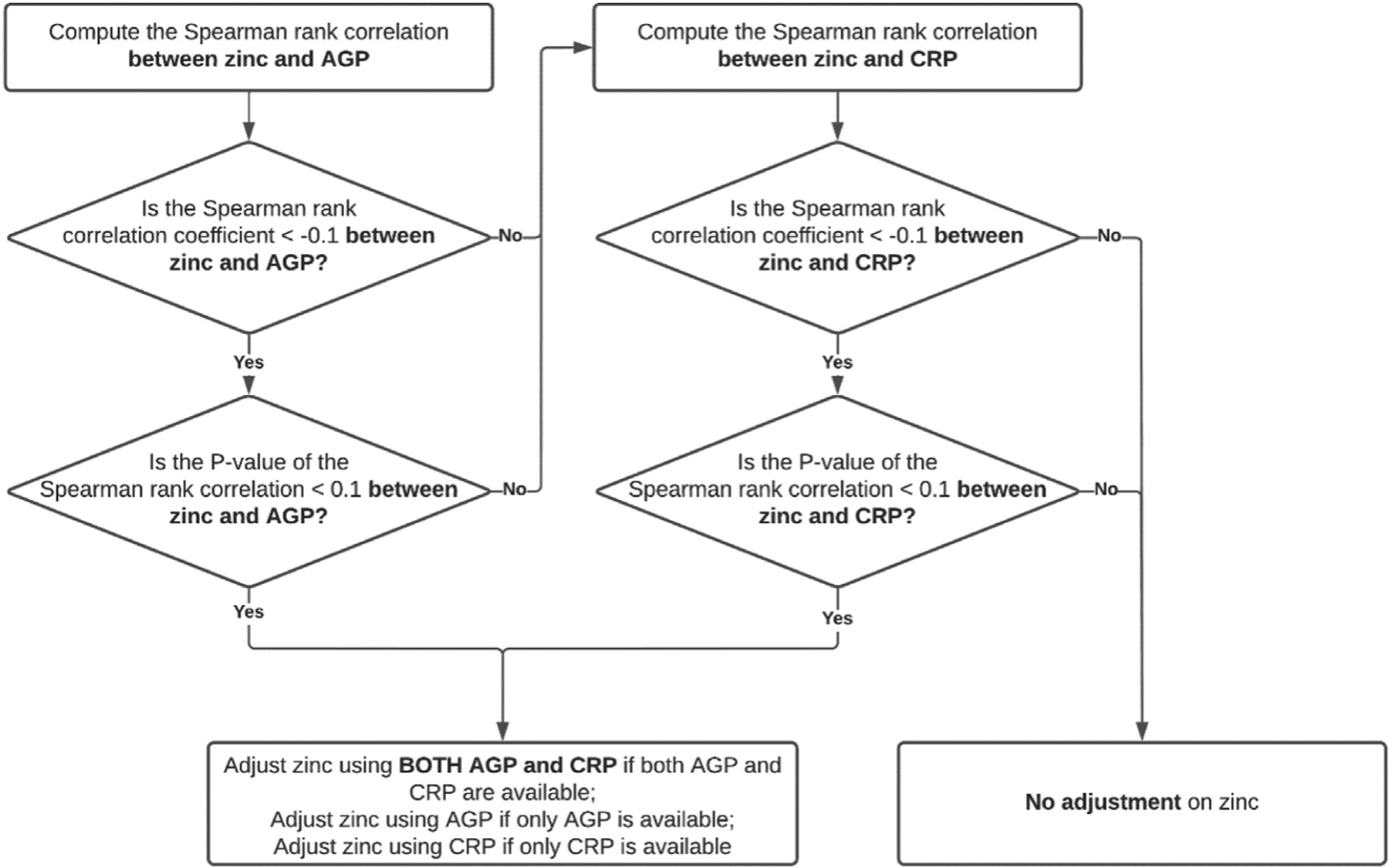
A decision-making tree on whether serum zinc should be adjusted for inflammation in preschool-age children. We recommend checking the decile plots of serum zinc and AGP/CRP as the first step before using this figure to determine whether serum zinc should be adjusted. AGP, α-1-acid glycoprotein.

The BRINDA inflammation adjustment method should only be used when there is both biological and statistical evidence of a relationship between micronutrient biomarkers and inflammation markers in the population being studied. It should not be applied indiscriminately to populations with high or low levels of inflammation without first establishing such a relation. Further, evidence suggests that inflammation may confound micronutrient biomarkers even in settings with low infection burden [18]. Based on current evidence in WRA and PSC groups, not all micronutrient biomarkers require adjustment for inflammation, nor do they need to be adjusted by both AGP and CRP. A schematic for when micronutrient biomarkers should be adjusted by which inflammation marker(s) is summarized in Figure 1. For example, serum and RBC folate and vitamin B-12 do not need to be adjusted for inflammation in either WRA or PSC [12], whereas sTfR should only be adjusted for AGP (but not CRP) [10] in both groups. Serum zinc does not routinely need adjustment for inflammation in WRA [11]. However, in PSC, serum zinc should be adjusted by both AGP and CRP only if Spearman correlation between serum zinc and either AGP or CRP is a negative (*r* < −0.1) and marginally significant (*P* < 0.1) [11]. A decision tree on when serum zinc should be adjusted is presented in Figure 2. If only one inflammation biomarker is available for micronutrient biomarkers that need to be adjusted by both AGP and CRP, it is still recommended to use that inflammation marker to adjust for inflammation.

### Procedure of adjusting micronutrient biomarkers for inflammation

**FIGURE 3.**
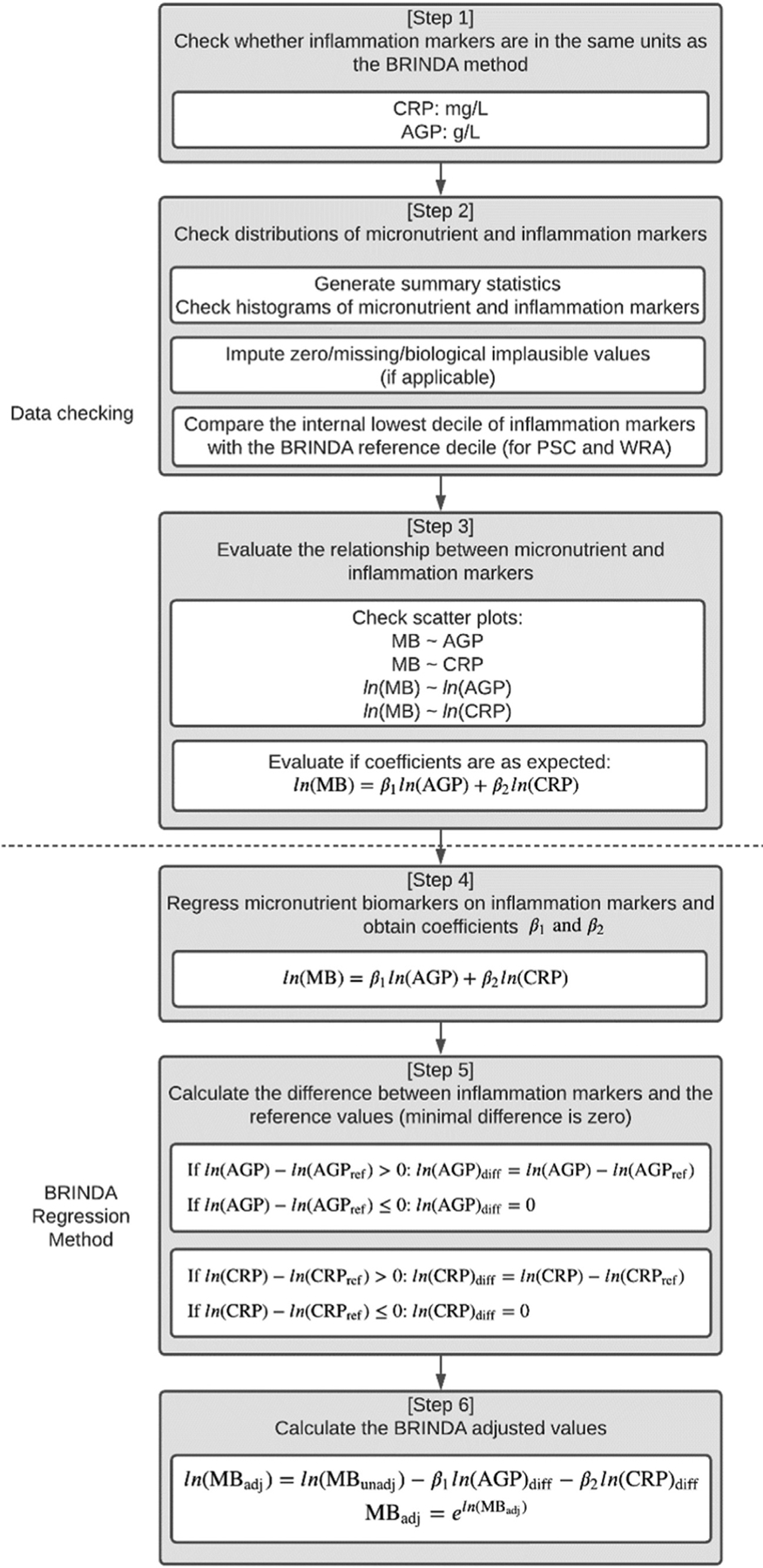
Procedures of checking data and applying the BRINDA adjustment method for micronutrient biomarkers that require adjustment for inflammation. The relation between iron and inflammation markers should be positive, whereas the relation between micronutrient biomarkers such as vitamin A or zinc and inflammation markers should be negative. For soluble transferrin receptor, only AGP should be included in the BRINDA adjustment; for zinc, adjustment using both AGP and CRP is needed only if there is a negative (*r* < −0.1) and marginally significant (*P* < 0.1) Spearman correlation between serum zinc and either AGP or CRP in PSC. AGP, α-1-acid glycoprotein; MB, micro-nutrient biomarkers; PSC, preschool-age children; WRA, women of reproductive age.

For micronutrient biomarkers requiring adjustment for inflammation, the procedure for applying the BRINDA inflammation adjustment method is illustrated in 6 steps in Figure 3. Steps 1 through 3 provide guidance on data checks and determining whether there is a correlation between micronutrient biomarkers and inflammation. Even though BRINDA has provided general guidance on which micronutrient biomarkers should be adjusted and by which inflammation marker(s) through publications [8–10, 12] including this paper, users are still advised to follow Steps 1 through 3 to check their own data for a relation between micro-nutrient and inflammation markers before applying for the BRINDA inflammation adjustment method. If such a relation does not exist, users are not advised to continue with BRINDA inflammation adjustment. Steps 4 through 6 describe how to apply the BRINDA inflammation adjustment method. For sTfR, where only AGP is needed to adjust for inflammation, users should just focus on evaluating the relation between AGP and sTfR.

- **Step 1**. Users should check the unit of inflammation markers. The units of AGP and CRP should be in g/L and mg/L, respectively. For micronutrient biomarkers, there is no specific unit requirement, and the units of adjusted micronutrient biomarkers will be the same as those of the unadjusted micronutrient bio-markers. However, it is recommended to report micronutrient biomarkers using either the International System of Units or conventional units. It is important to note that when calculating micronutrient deficiencies, the units of the biomarkers should match the units of the cut-off values used.
- **Step 2**. Users should generate univariate summary statistics of the micronutrient and inflammation markers and visualize their data by plotting the distributions of micronutrient and inflammation markers. Since both micronutrient and inflammation biomarkers will be natural log-transformed, it is important for users to pay attention to values that are clustered around the limits of detections (LoDs) and 0. These values may affect the BRINDA inflammation adjustment results, as values outside the LoDs may be recorded as 0 or the LoD. If any biomarkers have 0, missing, or biologically implausible values, it is recommended to conduct appropriate imputation. There are various imputation methods that can be used, from simply excluding values outside of the LoDs or biologically implausible values, to conducting more sophisticated multiple imputations [19]. Gosdin et al. [20] recommended using random number single imputation for CRP values below lower LoDs. Users can also try using different imputation methods and evaluate whether they can lead to different estimates of the prevalence of micronutrient deficiencies using BRINDA inflammation-adjusted values. If different imputation methods yield the same estimates of micronutrient deficiencies, users can use any of the methods. However, if differences exist between estimates from different imputation methods, users may want to conduct a deeper analysis to understand the underlying cause of the differences. This could involve carrying out additional laboratory analysis on the samples, conducting further research and analysis, or seeking input from experts in the field to identify potential factors that could be contributing to the observed differences.
  Users should also compare the internal lowest decile (i.e., 10%) of AGP and CRP with the BRINDA external reference decile value for PSC (AGP: 0.59 g/L and CRP: 0.10 mg/L) and WRA (AGP: 0.54 g/L and CRP: 0.16 mg/L) groups [4]. If AGP and CRP deciles from the users’ own analysis are substantially different (i.e., more than 20%) from the BRINDA external reference values, users should use internal deciles generated from their datasets instead of the BRINDA external AGP and CRP deciles.
- **Step 3**. Users should evaluate whether the bivariate relation between micronutrient and inflammation markers is as expected. Users can carry out scatter plots between micronutrients and AGP or CRP, as well as in their logarithm forms. Users can also fit the linear regression model of micronutrient biomarkers on AGP and CRP, all in logarithm forms. The relation between ferritin or sTfR and inflammation markers should be positive, whereas the relation between other micronutrient biomarkers like vitamin A or zinc and inflammation markers should be negative. If the expected relation between micronutrient and inflammation markers does not exist or displays the opposite direction, users are not advised to continue with steps 4 through 6 to perform the BRINDA inflammation adjustment method.
- **Step 4**. The first step to applying the BRINDA inflammation adjustment method is to conduct an unweighted regression analysis of micronutrient biomarkers on both AGP and CRP, instead of AGP and CRP individually and obtain the estimated β coefficients for AGP and CRP.
- **Step 5**. Users need to calculate the difference between inflammation markers and the external reference decile value. For each observation, if ln(AGP) is greater than the logarithm of BRINDA’s external AGP value, the difference is calculated as ln(AGP) minus ln(AGP reference value). If ln(AGP) is smaller than or equal to the logarithm of BRINDA’s external AGP value, the difference is 0, which means no adjustment is needed for observations with AGP below the threshold. For example, in a PSC dataset, if AGP is 2.1 for one child, the difference is ln(2.1) – ln(0.59) = 1.27; if AGP is 0.3 for another child, the difference is 0 because ln(0.3) is smaller than ln(0.59). The same logic applies to CRP.
- **Step 6**. Users can calculate the logarithm of adjusted micro-nutrient biomarker values for inflammation using equation 1. The adjusted micronutrient values are based on the unadjusted micronutrient values, estimated coefficients, and the difference between the inflammation marker and AGP and CRP reference value.
(1) $${\text{ln(MB}}_{\text{adj}}{\text{) = ln(MB}}_{\text{unadj}}\text{) }-\;\beta_{\text{1}}\times \text{ln}{\left(\text{AGP}\right)}_{\text{diff}}-\beta_{\text{2}}\times \text{ln}{\left(\text{CRP}\right)}_{\text{diff}}$$
  Users then can obtain the BRINDA inflammation-adjusted micronutrient biomarker values by exponentiating the results from equation 1.

## Special Consideration for Malaria

**FIGURE 4.**
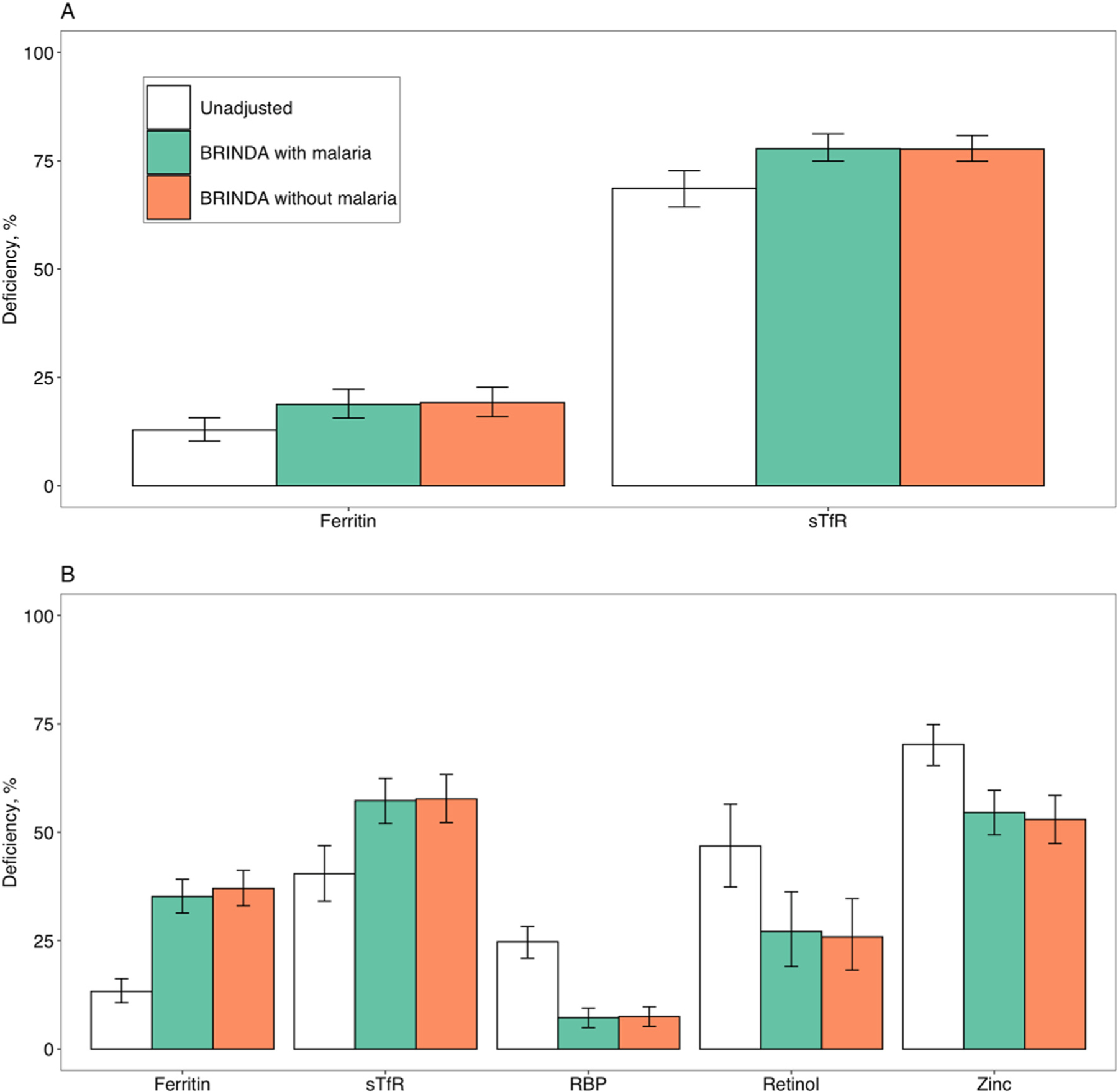
Comparison of the prevalence of micronutrient deficiencies using the BRINDA method with and without malaria in (A) WRA and (B) PSC. Five datasets in WRA (n = 4768) and 8 datasets in PSC (n = 6662) for ferritin, 5 datasets in WRA (n = 4768) and 7 datasets in PSC (n = 6251) for sTfR, 7 datasets in PSC (n = 6251) for RBP, 3 datasets in PSC (n = 1023) for serum retinol, and 2 datasets in PSC (n = 1841) for zinc were used in the analysis. Error bars show prevalence ± standard error. RBP, retinol binding protein; sTfR, soluble transferrin receptor.

In BRINDA’s initial publication [7], malaria status was added as a covariate in the BRINDA inflammation adjustment method and was also included in the WHO ferritin guideline [14]. Later BRINDA analysis showed that including malaria only altered the adjusted values of retinol and RBP slightly [8,9]. In this study, the role of malaria as a binary variable on a series of micronutrient biomarkers was evaluated using the latest BRINDA database. Five datasets in WRA (n = 4768) and 8 datasets in PSC (n = 6662) for ferritin, 5 datasets in WRA (n = 4768) and 7 datasets in PSC (n = 6251) for sTfR, 7 datasets in PSC (n = 6251) for RBP, 3 datasets in PSC (n = 1023) for serum retinol, and 2 datasets in PSC (n = 1841) for zinc were used in the analysis (data source is shown in Supplementary File 1). Overall, the median prevalence of iron deficiency in WRA was very similar in comparing the BRINDA adjustment method with and without malaria terms (ferritin: 18.8% compared with 19.2%; sTfR: 77.8% compared with 77.7%). Similarly, in PSC, when estimating iron deficiency using ferritin and sTfR, vitamin A deficiency using RBP and retinol, and zinc deficiency using serum zinc, the prevalence estimates of micronutrient deficiencies were similar using the BRINDA inflammation adjustment method with or without malaria as a binary variable (Figure 4). Therefore, adding malaria as a binary variable in the BRINDA inflammation adjustment for biomarkers of iron, vitamin A, and zinc is not necessary.

## BRINDA Inflammation Adjustment R Package and SAS Macro

The BRINDA inflammation adjustment R package and SAS macro are user-friendly, all-in-one statistical analysis software that facilitate the application of the BRINDA adjustment method for 5 micronutrient biomarkers, namely, serum RBP, retinol, ferritin, sTfR, and zinc (when appropriate), using AGP and CRP for various population groups. Because it is not recommended to adjust for inflammation for serum and RBC folate and vitamin B-12, the package and macro do not include those micronutrient biomarkers. The BRINDA inflammation adjustment R package and SAS macro have 3 main functions: *1*) evaluating whether applying the BRINDA adjustment method is needed for certain micronutrient biomarkers based on previous publications (Figure 1); *2*) determining whether zinc needs adjustment based on the correlation between zinc and inflammation markers from users’ data (Figure 2); and *3*) applying the BRINDA inflammation adjustment method (Figure 3 steps 4 to 6).

The BRINDA inflammation adjustment R package and SAS macro do not include functionality for data checks nor determining the statistical relation between micronutrient biomarkers and inflammation for user’s data. These steps (Figure 3 steps 1–3) require visualization and research-specific knowledge, such as information on LoDs and laboratory methods, which are beyond the scope of the package or macro. Users are responsible for completing these steps prior to applying the BRINDA inflammation adjustment R package and SAS macro to their data.

An overview of inputs, core content, and outputs for the BRINDA inflammation adjustment R package and SAS macro are illustrated in Supplementary File 2. The training materials and video tutorials for using the package and macro are available on the BRINDA website (https://www.brinda-nutrition.org/the-brinda-approach) [21,22]. In short, users need to specify the *1*) name of their dataset, *2*) variable names of the micronutrient and inflammation markers, *3*) population group of the dataset, *4*) user specified AGP and CRP reference values (which can be input only if the population group is “manual”), and *5*) output format. After a successful run, the BRINDA inflammation adjustment package will generate a new dataset containing additional variables of adjusted micronutrient biomarkers (by default). If users specify the output format as “full,” the output dataset will also include additional variables such as coefficients of regressions of micronutrient biomarkers on AGP and CRP and natural logs of AGP/CRP reference values. It is of note that the BRINDA software allows users to input one inflammation marker (AGP or CRP) instead of both inflammation markers and provides the adjusted micronutrient biomarker values accordingly.

## Discussion

This paper provides current and practical guidance on applying the BRINDA inflammation adjustment method, and the BRINDA inflammation adjustment R package and SAS macro provide a streamlined structure that can shorten the time spent applying the BRINDA method.

Addressing the role of inflammation in assessing micro-nutrient status is essential to interpret micronutrient deficiencies correctly [14]. The BRINDA method has been used in various studies and national surveys [23–25] and included in WHO ferritin guidelines [14] and CDC’s Micronutrient Manual and Toolkit [15]. The evidence gained from the BRINDA project has provided important insights to research, policy, and programming [26]. The role of malaria as a binary variable does not affect the results of BRINDA inflammation adjustment on a series of micronutrient biomarkers, namely, ferritin, sTfR, retinol, RBP, and zinc, using the latest BRINDA database. However, the stage of malaria infection may influence the results for the BRINDA inflammation adjustment method [24] and warrants more consideration in the future. Although the BRINDA inflammation adjustment method has not been applied within clinical settings, research on its utility in this setting is warranted.

In this paper, the open-access BRINDA inflammation adjustment R package and SAS macro were introduced. The package and macro, available sample code, video, and training materials [21,22] can further enhance the use of the BRINDA inflammation adjustment methods by a diverse audience and can shorten the time between data collection and interpretation. Although similar packages for STATA and SPSS software have not been developed, an interactive web portal on the BRINDA inflammation adjustment method is being developed. This can allow users to apply the BRINDA inflammation adjustment without the use of any software. Users will *1*) upload their data to an encrypted website portal and *2*) select the variable names of micronutrient and inflammation markers and desired cut-off values, and then BRINDA inflammation-adjusted micronutrient values and a summary of micronutrient deficiencies will be downloaded.

In conclusion, this paper provides clear guidance and introduces the user-friendly R package and SAS macro to apply the BRINDA inflammation adjustment method, which can facilitate micronutrient biomarker analysis for research and to inform nutrition policies and programs.

## Supplementary Material

Supplementary appendix

## Abbreviations used:

AGP: α-1-acid glycoprotein
BRINDA: Biomarkers Reflecting Inflammation and Nutritional Determinants of Anemia
LoD: limit of detection
PSC: preschool-age children
RBP: retinol binding protein
sTfR: soluble transferrin receptor
WRA: women of reproductive age
