# Supplementary material for: A Practical Guide to Adjust Micronutrient Biomarkers for Inflammation Using the BRINDA Method
Source: J Nutr. Author manuscript; Available in PMC 2023 May 22. (PMC10202121; doi:10.1016/j.tjnut.2023.02.016)
Supplement: Supplementary appendix [file NIHMS1898093-supplement-Supplementary_appendix.docx]

**Online Supplementary Material 1**

**Datasets used in “special consideration for malaria” section^1^**

| **Dataset (year)** | **Dataset name** | **Ferritin** | **sTfR** | **RBP** | **Retinol** | **Zinc** | **Reference** |
| --- | --- | --- | --- | --- | --- | --- | --- |
| Cote d’Ivoire (2007) | Cote D’Ivoire multiple indicator cluster survey 2006 | WRA, PSC | WRA, PSC | PSC |  | PSC | (1) |
| Cameroon (2009) | Cameroon National Micronutrient Survey | WRA, PSC | WRA, PSC | PSC | PSC |  | (2) |
| Kenya (2007) | Nyando Integrated Child Health and Education Project (NICHE) baseline | PSC | PSC | PSC |  |  | (3) |
| Kenya (2010) | Nyando Integrated Child Health and Education Project (NICHE) | PSC | PSC | PSC |  |  | (4) |
| Liberia (2011) | Liberia National Micronutrient Survey 2011 | WRA, PSC | WRA, PSC | PSC |  |  | (5) |
| Malawi (2016) | Malawi Demographic and Health Survey 2015-2016 | WRA, PSC | WRA, PSC | PSC |  | PSC | (6) |
| Nigeria (2012) | 2011 Akwa Ibom State Nutrition Survey | WRA, PSC | WRA, PSC | PSC | PSC |  | (7) |
| Zambia (2009) | Nutrition Survey in Central and Eastern Provinces, Zambia 2009 | PSC |  |  | PSC |  | (8) |

^1^PSC, Preschool-age Children; RBP, Retinol Binding Protein; sTfR, soluble Transferrin Receptor; WRA, Women of Reproductive Age.

**References:**

(1) Rohner, F.; Tschannen, A. B.; Northrop-Clewes, C.; Kouassi-Gohou, V.; Bosso, P. E.; Mascie-Taylor, N. Comparison of a Possession Score and Poverty Index in Predicting Anaemia and Undernutrition in Pre-School Children and Women of Reproductive Age in Rural and Urban Cote d’Ivoure. *Public Health Nutrition* **2012**, *15* (9), 1620–1629.

(2) Engle-Stone, R.; Ndjebayi, A. O.; Nankap, M.; Brown, K. H. Consumption of Potentially Fortifiable Foods by Women and Young Children Varies by Ecological Zone and Socio-Economic Status in Cameroon. *The Journal of Nutrition* **2012**, *142* (3), 555–565.

(3) CDC. *Baseline Data from the Nyando Integrated Child Health and Education Project - Kenya 2007*; 2007. <https://www.cdc.gov/mmwr/preview/mmwrhtml/mm5642a4.htm>.

(4) Foote, E. M.; Sullivan, K. M.; Ruth, L. J.; Oremo, J.; Sadumah, I.; Williams, T. N.; Suchdev, P. S. Determinants of Anemia among Preschool Children in Rural, Western Kenya. *Am J Trop Med Hyg* **2013**, *88* (4), 757–764.

(5) UNICEF; Liberia Institute for Statistics and Geo-information Services (LISGIS); Government of Liberia. *Liberia National Micronutrient Survey 2011*; 2011. <http://ghdx.healthdata.org/record/liberia-national-micronutrient-survey-2011>.

(6) National Statistics Office (NSO); ICF. *Malawi Demographic and Health Survey 2015-2016*; 2016. <https://dhsprogram.com/pubs/pdf/FR319/FR319.pdf>.

(7) Maziya-Dixon, B. B.; Sanusi, R. A.; Oguntona, E. B. *Anthropometry and Biochemical Assessments: Women of Childbearing Age and Children 6-59 Months of Age from Rural Areas in Akwa Ibom State, Nigeria*; 2012. <https://www.iita.org/wp-content/uploads/2021/01/Anthropometry-and-Biochemical-Assessments-of-Women-of-Childbearing-Age-and-Children-6%E2%80%9359-Mths-of-Age-from-Rural-Areas-in-Akwa-Ibom-State-Nigeria.pdf>.

(8) Hotz, C.; Palaniappan, U.; Chileshe, J.; Kafwembe, E.; Siamusantu, W. *Nutrition Survey in Central and Eastern Provinces, Zambia 2009: Focus on Vitamin A and Maize Intakes, and Vitamin A Status among Women and Children*; 2011.

**Online Supplementary Material 2**

**BRINDA R package and SAS macro**

**User Manual**

Overview of inputs, core content, and outputs for Biomarkers Reflecting Inflammation and Nutritional Determinants of Anemia (BRINDA) R package and SAS macro is illustrated in **Supplementary Fig. 1**. Users need to specify 1) the name of their dataset, 2) variable names of micronutrient and inflammation markers, 3) population group of the dataset (women of reproductive age (WRA), preschool-age children (PSC), other, or manual), 4) user-specified α-1-acid glycoprotein (AGP) and C-Reactive Protein (CRP) reference values (allowed to input only if the population group is “manual”), and 5) output format (simple or full; simple by default). After users input all information, the BRINDA R package will proceed with the following four steps:

**Step 1:** The BRINDA R package will carry out checks and provide guidance to correct errors (if errors occur).

**Step 2:** After the inputs and dataset are error-free, the BRINDA R package will generate the AGP and CRP reference values based on users’ inputs:

- If the population group is WRA, the reference value of ln(AGP) is -0.52, and the reference value of ln(CRP) is -2.26;
- If the population group is PSC, the reference value of ln(AGP) is -0.62, and the reference value of ln(CRP) is -1.83;
- If the population group is other, the BRINDA R package will first calculate the lowest decile of AGP and CRP and take natural logs of these values as the reference values;
- If the population group is manual, the BRINDA R package will use natural logs of user-specified AGP and CRP as the reference values.

**Step 3:** The BRINDA package will adjust micronutrient biomarkers based on the relationship between micronutrient biomarkers and inflammation markers and the reference AGP and CRP values. Please note that the BRINDA package allows users to input one inflammation marker (AGP or CRP) instead of both inflammation markers and provides the adjusted micronutrient biomarker values accordingly.

**Step 4:** After a successful run, the BRINDA package will generate a new dataset (by default) containing additional variables of adjusted micronutrient biomarkers. If users specify output format as “full”, the output dataset will also include additional variables such as coefficients of regressions of micronutrient biomarkers on AGP and/or CRP, natural logs of AGP and/or CRP reference values, etc.

Supplementary figure 1: Overview of the BRINDA package inputs, core contents, and outputs


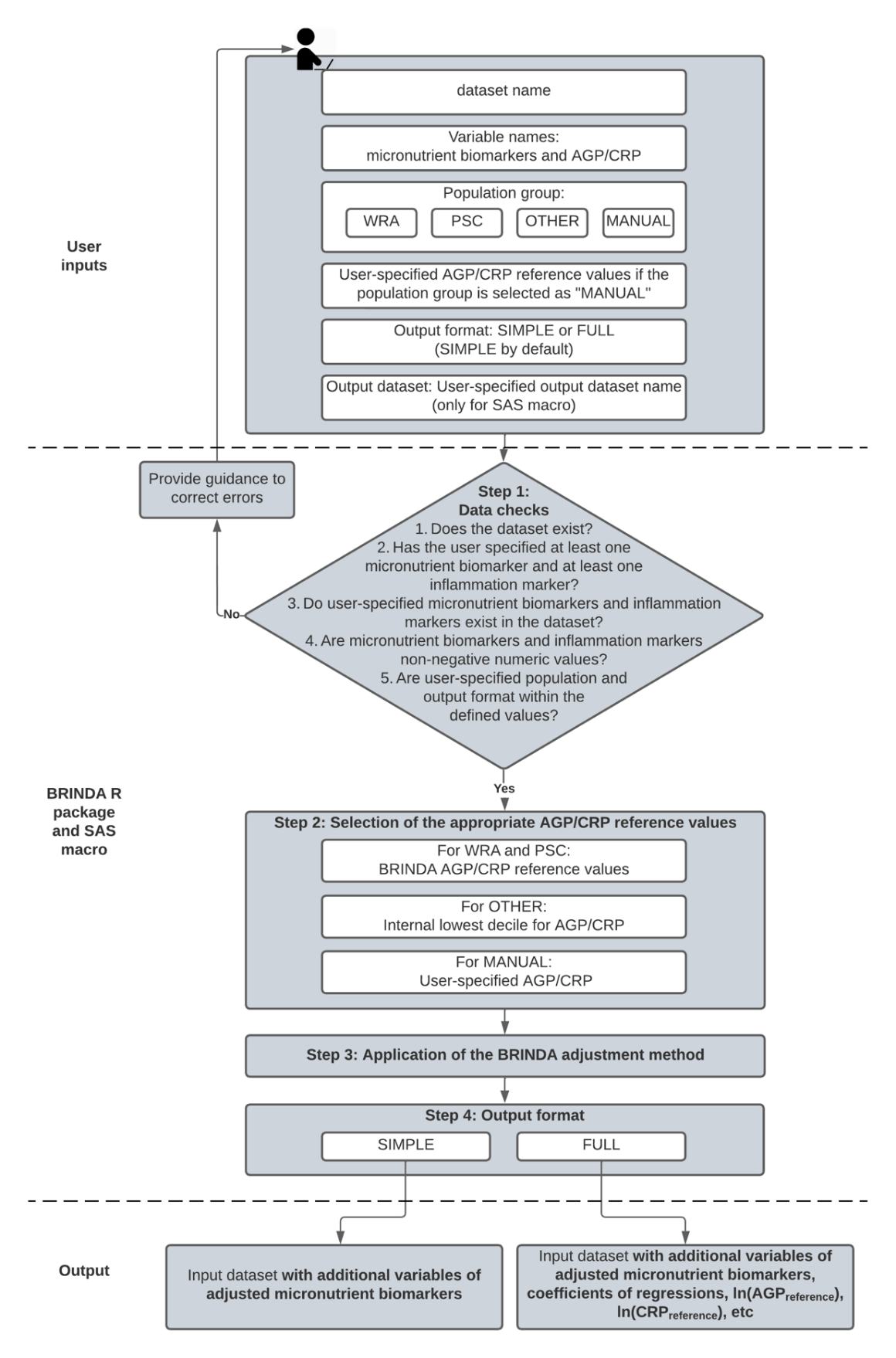


AGP, α-1-acid glycoprotein; CRP, C-Reactive Protein; PSC, Preschool-age Children; WRA, Women of Reproductive Age.
